# Psychosocial safety climate and self-efficacy: Moderating role of job-related expectations in Pakistani private-sector employees during the COVID-19 pandemic

**DOI:** 10.3389/fpsyg.2022.1016050

**Published:** 2023-02-28

**Authors:** Saira Maqsood, Marva Sohail, Fatima Naeem, Mohammad Nazri, Deep Fatima

**Affiliations:** ^1^Department of Psychology, Lahore Garrison University, Lahore, Pakistan; ^2^Faculty of business and economics, Department of Management, University of Malaya, Kuala Lumpur, Malaysia

**Keywords:** organizational climate, psychosocial safety, self-efficacy, COVID-19, job demands, employees

## Abstract

The labor force in Pakistan comprises 59.8 million individuals. The employees have faced major changes in work dynamics and psychosocial safety climate during the COVID-19 pandemic. The purpose of the current study is to find the relationship between psychosocial safety climate, self-efficacy, and job-related expectations. It explores the moderating role of job-related expectations on the relationship between psychosocial safety climate and self-efficacy. It was hypothesized that there is likely to be a significant relationship between psychosocial safety climate, self-efficacy, and job-related expectations, job-related expectations are likely to moderate the relationship between psychosocial safety climate and self-efficacy, and there are likely to be differences between married and unmarried employees; men and women; satisfied and unsatisfied employees with respect to psychosocial safety, self-efficacy, and job-related expectations. A correlational research design and a convenience sampling strategy were used. A total of 281 employees (*M* = 30.74 years, SD = 10.99) of the private-sector (including educational, industrial, and IT) organizations during the COVID-19 pandemic were part of the study. Results show that psychosocial safety climate had a positive significant relationship with job-related expectations and self-efficacy. Job expectations also significantly correlated with self-efficacy. There were significant differences in measures of study variables with respect to gender, marital status, and employee satisfaction. This research has implications for administration, managers, policymakers, and organizational psychologists.

## 1. Introduction

The labor force in Pakistan comprises 59.8 million individuals, which break down into 1.4% employers, 43% employees, 36% entrepreneurs or self-employed individuals, and 20% unpaid family workers. Out of these, only 28% of employees working in the formal non-agricultural sector are protected by labor laws (Ahmad, 2020). Here, owing to a lack of proper remuneration and lower levels of job satisfaction, a major shift toward private organizations from public organizations is seen (Mitra, 2019). This shift is a testament to a major difference between public and private organizations which is employee behavior. The spectrum of employee behavior is due to personal as well as environmental factors. The difference in employee behavior at the workplace could be explained by organizational climate–a construct that is hard to explain as it is dependent upon the perception of employees (Berberoglu, 2018). Despite this, organizational climate is defined as the psychological and social characteristics and interaction among different groups working in an organization, which is influenced by management styles (Popa, 2011). It also illustrates an organization’s experiences, beliefs, morals, psychology, ideals, ethics, values, and behaviors (Hussain and Yousaf, 2011). The most common issues that affect organizational climate in recent times are a lack of flexibility and an innovative environment at work, which is caused by downsizing, economic constraints, and changes in work dynamics due to outsourcing (Permarupan et al., 2013; Berberoglu, 2018).

Organizational climate is further dramatically influenced due to COVID-19 as it brought with it socioeconomic shocks (Kniffin et al., 2021). Similar to the global economy, the Pakistani economy is also badly hit by the pandemic, especially the service sector. As per a report by Pakistan Worker’s Federation (2020), self-employed individuals along with the education, tourism, transport, and hospitality sectors are hardest hit. Additionally, nearly 25,000 expatriates have been laid off owing to the economic recession policies during the COVID-19 pandemic. The Centre for Labor Research estimated job disruptions for around 21 million workers in the country. A total of 10.5 million workers faced temporary unemployment as a result of preventive measures like provincial and national lockdowns (Rana, 2020). Employees who still had their jobs faced varying conditions including increased job demands, supply chain disruptions, lower consumer demands (Shafi et al., 2020), and pay cuts due to variances in the market (Aftab et al., 2021).

The additional threat was posed to employees’ health owing to the nature of the job (Prochazka et al., 2020) and public commuting coupled with a threat to the psychosocial safety climate at work (Seddighi et al., 2022). Psychosocial safety climate is a domain of organizational climate that is inclusive of the psychological safety and health of employees, which influences job design and sociorelational aspects of the work environment (Cox and Cheyne, 2000; Loh et al., 2020). It is a novel construct that is defined as policies, practices, and procedures for workers’ psychological health and safety (Dollard et al., 2017). Generally, the levels of psychosocial climate are lower than that of physical safety climate as organizations usually prioritize the latter (Idris et al., 2012; Platania et al., 2022). Psychosocial safety climate is a multifaceted construct comprising organizational commitment, management prioritization, and commitment along with the participation of employees and management in the prevention of stress (Dollard and Bakker, 2010; Brunetto et al., 2021). In organizations, stress stems from the work environment, work schedule, work pace, and job content (Uronen et al., 2017).

In conditions like COVID-19 that have an element of shock, the psychosocial safety climate holds a crucial value as it can help build resilient workplaces (Dollard and Bailey, 2021) and change the practices of management (Teoh and Kee, 2020). The psychosocial factors can further influence an employee both positively, influencing work processes, development pathways, platforms, job security, and safety, as well as negatively, influencing salary deduction, limited job control, and job insecurity (Rus and Buzarna-Tihenea, 2014). Furthermore, worker attitude, leadership, and strategy lead management to ensure a better psychosocial safety climate (Elfi, 2020) that also serves as a precursor for individual as well as team motivation (Hu et al., 2022) and success (Raja et al., 2019).

On joining an organization, an employee has some expectations related to the job. These expectations can be related to remuneration, work flexibility, work conditions, professional development, and working hours (Mirabela et al., 2016). Irrespective of the psychosocial safety climate, these expectations also influence personal and job-related outcomes. Job-related expectations can further relate to available job demands and resources that underlie the psychosocial safety climate (Afsharian et al., 2018).

One of the outcomes related to the psychosocial safety climate domain of organizational climate is the self-efficacy of the employees. It is characterized by a belief of an individual related to their capability to act in a certain manner (Bradley et al., 2017) to reach their goals (Bandura, 1977) and the willingness to persevere on the way (Bradley et al., 2017). Five main characteristics utilized by individuals to increase self-efficacy through regulation and sustaining the behavior include symbolizing, forethought, observational, self-regulatory, and self-reflective (Stajkovic and Luthans, 2003). Higher levels of self-efficacy improve the overall productivity of the organization as, on completion of the tasks, employees engage in a self-fulfilling cycle necessitating the management to create a holistic environment and better organizational climate for the sake of the betterment of employees as well as the organization (Lyons and Bandura, 2019).

Previous research has discussed the direct relationship between the variables under study, i.e., psychosocial safety climate, self-efficacy, and job-related expectations. The model discussing the moderating role of job-related expectations in the relationship between psychosocial safety climate and self-efficacy has not been explored in any of the research, especially in the COVID-19 scenario. Considering the pandemic situation changed, the work dynamics and the job-related expectations along with the psychosocial safety climate were affected greatly. The exploration in current research would facilitate understanding the relationship and effect of variables in an emergency or unprecedented situation like the pandemic.

The current study aims to find the relationship between psychosocial safety climate, self-efficacy, and job-related expectations. It studies the predictive role of psychosocial safety climate and job-related resources on self-efficacy. It explores the moderating role of job-related expectations on the relationship between psychosocial safety climate and self-efficacy. It also explores the difference between married and unmarried employees with respect to study variables. This holds importance during the COVID-19 pandemic as it has shaken the work and organizations to the core. Organizations have shifted from thriving to survival mode which has changed dynamics, and the nature of work has changed. Work has shifted from physical to online or hybrid modes, changing the domains of organizational climate, associated factors, expectations related to the job as well as the resultant self-efficacy. The research particularly the literature section uses the broader term of organizational climate to refer to psychosocial safety climate, which is its subdomain, as most of the literature utilizes the aforementioned term to refer to all the subdomains.

## 2. Literature and hypotheses development

### 2.1. Theoretical framework

Social cognitive career theory (Lent, 2013) and conservation of resources (COR) theory (Hobfoll, 1989) provide the theoretical support for this research. According to the social cognitive career theory, self-efficacy is associated with three factors, i.e., environmental factors, outcome expectations, and personal goals. It is pertinent to note here that self-efficacy is dynamic and keeps on changing. In this study, the environmental factor that is being considered is psychosocial safety climate. The personal goal derivative that is being studied in this research is job-related expectations. The outcome expectations prong of the model corresponds to both the factors that are being studied in this study, i.e., psychosocial safety climate and job-related expectations. This lays foundation for testing direct relationship between the variables. Employees tend to get involved in activities that have positive outcomes and adjust levels of self-efficacy accordingly. Therefore, activities and behavior that contribute toward a better psychosocial safety climate and fulfill their job expectations increase the chances of repetition of the activity, which depicts higher self-efficacy. Employees are likely to be satisfied depending upon the extent to which job expectations are met. These expectations include job control, career progression, and personal and professional development. The job-related expectations, on the other hand, are influenced by environmental and personal factors. Individuals develop these expectations through social learning, i.e., observation and vicarious learning, which is explained through social learning theory (Bandura, 1962). In the case of Pakistani collectivist society, these factors are the influence of family, peers, society, and socioeconomic status.

Additionally, the COR theory (Hobfoll, 1989) lays the foundation for testing the moderating role of job-related expectations on the relationship between psychosocial safety climate and self-efficacy. Based on this theory, resources are of varying nature, ranging from objects, personal characteristics, and conditions to energies and can be generally divided into external and internal resources. However, internal and external resources are linked, and individuals invest available resources to gain more resources. The primary focus of the theory is on the loss and gain of resources. Psychosocial safety climate is a resource (external condition) that has a direct relationship with self-efficacy (internal energy). As per the second principle of COR theory, i.e., resource investments, resources must be invested to protect against resource loss, recover from resource loss, and gain resources. Coupled with the principle of theory and study by Brouer et al. (2011), resources are linked; hence, they influence the relationship that exists between other resources. Here, the job-related expectations (internal and external conditions) are the resources that influence the relationship that exists between psychosocial safety climate and self-efficacy. Hence, creating a moderating relationship.

### 2.2. Psychosocial safety climate and self-efficacy

Literature related to research on teachers has shown that there is no direct relationship between organizational climate and self-efficacy and that other variables are involved in this relationship (Jaafari et al., 2012). On the other hand, similar research has shown that organizational climate accounts for the changes in self-efficacy among employees in the educational sector (Tobin et al., 2006). A direct effect between organizational climate and self-efficacy has also been found, in which the former is responsible for a 23% variance in self-efficacy (Yi et al., 2008). Higher levels of self-efficacy have been found among employees who have a supportive and safe organizational climate (Reaves and Cozzens, 2018). Strain in environment leads to a negative influence on self-efficacy (von Suchodoletz et al., 2018). An increase in both organizational climate and self-efficacy has positive outcomes for the organizations (Patras et al., 2020). Furthermore, various factors such as environment, teamwork, management effectiveness, involvement, reward and recognition, competency, and commitment are found to be associated with organizational climate. All these factors are positively related to motivation to work and self-efficacy (Mahal, 2009; Zhang and Liu, 2010; Karantzas et al., 2016). These factors are also dimensions of job-related expectations.

Based on the aforementioned research, it is hypothesized as follows:

H1: There is likely to be a significant positive relationship between psychosocial safety climate, self-efficacy, and job-related expectations among private-sector employees during the COVID-19 pandemic.

### 2.3. Job-related expectations as moderator

Job-related expectations are likely to play a significant role in the relationship between organizational climate and self-efficacy. Notably, 90% of employees in a survey have reported a participatory work environment as a job-related expectation, whereas 80% of opportunities to learn have also been one of the top job expectations (Sharma and Chully, 2020). Common job expectations that influence job-related outcomes include rewards, job security (Linz and Semykina, 2013), good working environment, remuneration, career development (Čiarnienė et al., 2010), and work–life balance (Egerová et al., 2021). Unmet job-related expectations in areas of salary, level of interest and growth prospects (Zhang et al., 2019), workplace communication, management, and role conflict lead to poor identification of an organization (Čiarnienė et al., 2010), affecting its organizational climate (Schiff and Leip, 2018).

Job-related expectations have a significant relationship with self-efficacy (Maden et al., 2016). The subdomain of job expectations, i.e., professional development, influences an employee’s self-efficacy (Posnanski, 2017; An, 2018). In organizations in which professional development is a priority, employees are likely to have higher levels of self-efficacy (Martin et al., 2008; Tschannen-Moran and McMaster, 2009) and higher productivity levels. Employees have also shown a significant increase in their self-efficacy after workshops targeting professional development (Watson, 2006).

The subdomain of job expectations, i.e., compensation (Linden, 2015), also has a significant relationship with self-efficacy (Divandari et al., 2018). This implies that employees whose expectations related to remuneration are fulfilled have higher levels of self-efficacy (Hu et al., 2018). This expectation also moderates the effect on self-efficacy (Kim et al., 2008). Also, sociocultural factors like cultural norms at the community level (Hopp and Stephan, 2012), demographic characteristics (Mensah and Mi, 2017), generation, socioeconomic status, acculturation, and enculturation (Aguayo et al., 2011; Wennberg et al., 2013) influence self-efficacy. These sociocultural factors, on the other hand, play a role in developing job-related expectations, especially in collectivist cultures like Pakistan.

Based on the aforementioned research, it is hypothesized as follows:

H2: Job-related expectations are likely to moderate the relationship between psychosocial safety climate and self-efficacy among private-sector employees during the COVID-19 pandemic, in which an increase in the expectations would strengthen the relationship between psychosocial safety climate and self-efficacy.

### 2.4. Difference between variables with respect to demographic characteristics

Research has shown that individual factors such as gender, marital status, and demographics influence psychosocial safety climate scores (Zadow et al., 2019). It has been found that women have a lower psychosocial climate score as compared to their male counterparts (Berthelsen et al., 2020). In another instance, the relationship between psychosocial safety climate and psychological outcomes was more prominent in men as compared to women (Jane Zadow et al., 2021). Similarly, gender has an influence on job expectations and associated job satisfaction (Gasser et al., 2000; Sumner and Niederman, 2004). Women tend to have higher job expectations as compared to men, based on a study (Chullen et al., 2015). Exploring the gender differences in self-efficacy, it was found that there are significant gender differences with respect to self-efficacy (Singh and Udainiya, 2009). In one study, women were found to have higher self-efficacy in creative tasks, while men have higher self-efficacy in logical tasks (Huang, 2013). McKay et al. (2014) established that men have higher scores on social self-efficacy while women have higher scores on emotional self-efficacy.

The variables such as psychosocial safety climate, self-efficacy, and job-related expectations also tend to differ in individuals who are married and unmarried. This is due to societal demands, gender roles, and family responsibilities (Azim et al., 2013; Odanga et al., 2015; Ghafoor et al., 2020; Maqsood et al., 2021).

Job satisfaction and other psychological determinants of self-efficacy differ substantially in employees working physically and remotely as found in research during the COVID-19 pandemic (Brunelle and Fortin, 2021). Job satisfaction is found to have a direct link with psychosocial safety climate (Geisler et al., 2019). There is also a significant link between job satisfaction and job expectations (Kong et al., 2015; Nie and Sousa-Poza, 2017). Employees who are satisfied with their job tend to have their job expectations fulfilled (Gazioglu and Tansel, 2006; Čiarnienė et al., 2010; Oraman et al., 2011; Linz and Semykina, 2013; Huang and Gamble, 2015). Further job satisfaction levels are associated with the self-efficacy of employees (Bargsted et al., 2019) in traditional as well as evolving gig economies (Riani et al., 2022).

Based on the aforementioned research, it is hypothesized as follows:

H3a: There are likely to be differences between men and women with respect to psychosocial safety climate, self-efficacy, and job-related expectations among private-sector employees during the COVID-19 pandemic, in which men would have higher scores on psychosocial safety climate and women would have higher scores on job expectations and self-efficacy.

H3b: There are likely to be differences between married and unmarried employees with respect to psychosocial safety climate, self-efficacy, and job-related expectations among private-sector employees during the COVID-19 pandemic, in which unmarried would have higher scores on study variables.

H3c: There are likely to be differences between satisfied and unsatisfied employees with respect to psychosocial safety climate, self-efficacy, and job-related expectations among private-sector employees during the COVID-19 pandemic, in which unsatisfied employees would have higher scores on study variables.

## 3. Methods

### 3.1. Sample and procedure

The study was based on the correlational research design. Correlational research is a type of non-experimental research in which the researcher assesses the statistical relationship between the variables without controlling extraneous variables (Curtis et al., 2016). It aimed to investigate psychosocial safety climate, job-related expectations, and self-efficacy in 281 employees of the private-sector (including educational, industrial, and IT) organization during the COVID-19 pandemic when they were working remotely. The sample had a mean age range of 18–60 years, with *M* = 30.74, and SD = 10.99. The convenience sampling strategy was used because of the unavailability of participants due to the lockdown in major cities of Pakistan during the pandemic COVID-19 situation in the current research. The sample was collected from 9 different organizations in Lahore, Pakistan. A total of 316 survey questionnaires were distributed among employees, out of which 281 survey questionnaires were valid and 35 questionnaires were discarded. The sample size was selected using the ratio of the number of subjects (*N*) to the number of items (*p*), three to five subjects per item. The data were collected online through Google Forms, which is a free online software to create surveys, as a part of Google’s web-based apps suite, due to travel restrictions and SOPs of COVID-19. The link to questionnaires, along with written instructions, was disseminated through social media platforms and official WhatsApp groups.

Informed consent was provided by the participants at the beginning of online data collection and they were given the right to withdraw from the research at any time they desired. The research was designed, conducted, and reported in compliance with the American Psychological Association (APA) guidelines.

The sample comprises men (169) and women (112). There were managers (22), lecturers (9), supervisors (12), employees working between scales 9th and 18th (196), and others (42%). The sample included employees who qualified for intermediate (10%), graduation (41%), and postgraduation levels (49%). The sample is composed of both married (*n* = 172; 61%) and unmarried employees (*n* = 109; 39%). Employees living in both joint (*n* = 175; 62%) and nuclear family systems (*n* = 106; 38%) were selected. Those employees who were regular or on a contract basis were included in the study.

### 3.2. Data analysis

Data were entered in SPSS version 26. Statistical significance was set at a 0.05 level. The normality of the distribution was tested using skewness and kurtosis. The value of skewness for the age of participants is −0.41 (left skewed), marital status is 0.46, family system is 0.50, job satisfaction is 0.72, psychosocial safety climate is 0.86, professional development subscale is 0.46, compensation subscale is 0.44, user relation is 0.49, and self-efficacy is 0.09, depicting that distribution of all these variables is right skewed. These values of skewness elicit that data are normal as acceptable skewness values for normality of data are between −2 and +2 (Curran et al., 1996; Byrne, 2010; Hair et al., 2010). Subsequently, the values of kurtosis for the age of participants is 1.8, marital status is −1.79, family system is −1.75, job satisfaction is −0.80, psychosocial safety climate is 6.44, professional development subscale is −0.25, compensation subscale is 0.25, user relation is −0.20, and self-efficacy is 0.01. These values of kurtosis show that data are normal as kurtosis values between −7 and +7 correspond to normal data (Curran et al., 1996; Byrne, 2010; Hair et al., 2010).

Cronbach’s alpha is used to test the reliability. Sociodemographic variables were analyzed using frequencies, percentages, means, and standard deviation. To find the association between variables psychosocial safety climate, job-related expectations, and self-efficacy, a correlation analysis was carried out. Moderation analysis was carried out using PROCESS MACRO (Hayes, 2013). Bootstrapping was run over 1,000, an infinite number of replications. Bootstrapping is a hypothesis testing and effect size estimating approach that makes no assumption about sampling distribution and the shape of the variable distributions in statistics (Preacher and Hayes, 2004).

Moderation is defined as a relationship between an independent and a dependent variable and it changes values according to a moderator variable (Dawson, 2014). According to Hayes et al. (2017), PROCESS MACRO automatically provides mean-centering system of the independent and moderating variables, eliminates multi-collinearity, and verifies the significance of simple slope in detail (Choi and Jang, 2022).

To find the mean difference in psychosocial safety climate, job-related expectations, and self-efficacy between married and unmarried employees, a *t*-test was used.

### 3.3. Measures

#### 3.3.1. Psychosocial safety climate scale

It is a 12-item scale developed by Hall et al., 2010, which assesses psychosocial safety climate. It consists of a five-point Likert scale, ranging from 1 = “Strongly Disagree” to 5 = “Strongly Agree.” It has four subscales (with Cronbach’s alpha reliability), namely, Management commitment (0.88), Management priority (0.90), Organizational communication (0.77), and Organizational participation (0.80). Examples of items of the subscales are as follows: (1) In my workplace, senior management acts quickly to correct problems/issues that affect employees’ psychological health; (2) senior management shows support for stress prevention through involvement and commitment. The reliability of Cronbach’s alpha of the complete scale is 0.78.

#### 3.3.2. Job expectation questionnaire

It is a 12-item scale developed by Villa-George et al. (2011), which is used to measure job-related expectations of employees. It has a five-point Likert scale, ranging from 0 = “I never had this expectation” to 4 = “It was fully met.” Job expectation questionnaire (JEQ) has three dimensions (with Cronbach’s alpha reliability), i.e., professional development (0.68), the example of an item of the subscale professional development is: I hoped for fair treatment within the work team; Compensation (0.56) example of an item of this subscale is: I had the idea that my salary would match my dedication and the hours of work I carry out; and User relation with (0.72) and example of an item is: I expected to find respect and good manners in the interaction with clients/users. The reliability of Cronbach’s alpha of the complete scale is 0.81.

#### 3.3.3. General self-efficacy scale

It is a 10-item scale developed by Schwarzer and Jerusalem (1995), which is used to measure the levels of general self-efficacy in individuals. It has a four-point Likert scale, ranging from 1 = “Not all true” to 4 = “Exactly true.” Its Cronbach’s alpha reliability for the entire measure is 0.80. An example of an item of this scale is: I can always manage to solve difficult problems if I try hard enough.

## 4. Results

The results of Table 1 showed that there were 60% of women and 40% of men (*M* = 30.74, SD = 10.99) who took part in this research. Out of these, 61.2% were married and 39% were unmarried participants, with an educational background of FA (10.7%), graduation (40.6%), and postgraduation (48.6%). Most of the participants belonged to joint families (62.3%) and the remaining belonged to nuclear (37.7%) families. The number of dependents varied, with 44.1% having more than two dependents, 42% having more than six, and 14% having more than eight dependents. The respondents reported they are working on the managerial scale (7.8%), supervisory position (4.3%), lecturer (3.2%), working between scales 9th and 18th (70%), and others (15%). Moreover, 53% of respondents were satisfied with their job, 32.1% were not satisfied, and 15% of participants responded to some extent according to their qualifications. Meanwhile, 55.5% of respondents reported that they get upset quickly in critical situations, 36.7% cannot get easily upset, and 7.8% responded to some extent. Furthermore, 58.4% of respondents reported as they have strong decision-making power in difficult situations, 28.5% reported that they do not have the ability to make decisions, and 13.2% to some extent.

**TABLE 1:** Demographic characteristics of the participants (*N* = 281).

Variables	*f* (%)
**Gender**	
Female	112 (60.10)
Male	169 (39.90)
**Educational status**	
FA	30 (10.70)
Graduation	114 (40.60)
Postgraduation	137 (48.60)
**Marital status**	
Married	172 (61.20)
Unmarried	109 (38.80)
**Family system**	
Joint	175 (62.30)
Nuclear	106 (37.70)
**No. of dependents**	
More than 2	124 (44.10)
More than 6	118 (42.00)
More than 8	39 (14.00)
**In which scale/grade do you work?**	
Manager	22 (7.80)
Supervisor	12 (4.30)
Lecturer	9 (3.20)
Working between 9th and 18th scales	196 (70)
Others	42 (15)
**Are you satisfied with your job according to your qualification?**	
Yes	148 (53)
No	90 (32.10)
To some extent	42 (15.00)
**Do you get upset quickly in a difficult situation?**	
Yes	156 (55.50)
No	103 (36.70)
To some extent	22 (7.80)
**Can you make a difficult decision easily?**	
Yes	164 (58.40)
No	80 (28.50)
To some extent	37 (13.20)

All the tables and figures reported in the article show standardized values.

### 4.1. Correlation analysis

The results of the product-moment correlation are shown in Table 2. The analyses showed that psychosocial safety climate has a positive significant relationship with job-related expectations and self-efficacy. Correlation analysis also showed that job-related expectations are significantly and positively correlated with self-efficacy. This accepts the Hypothesis (H1).

**TABLE 2:** Descriptive statistics and intercorrelations for the study variables (*N* = 281).

Measure	1	2	3
Psychosocial safety climate	–	0.35***	0.45***
Job-related expectations		–	0.43***
General self-efficacy			–
*M*	36.59	25.56	26.08
SD	8.30	7.78	5.61

*N* = 281, ***p < 0.01.

### 4.2. Moderation analysis

**FIGURE 1:**
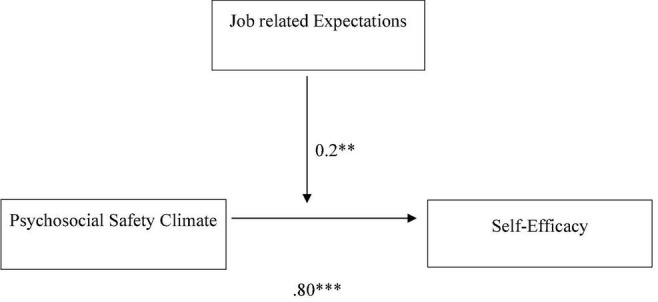
Moderation effects of job expectations on the relationship between psychosocial safety climate scale and self-efficacy. ^**^p < 0.01; ^***^p < 0.001.

**FIGURE 2:**
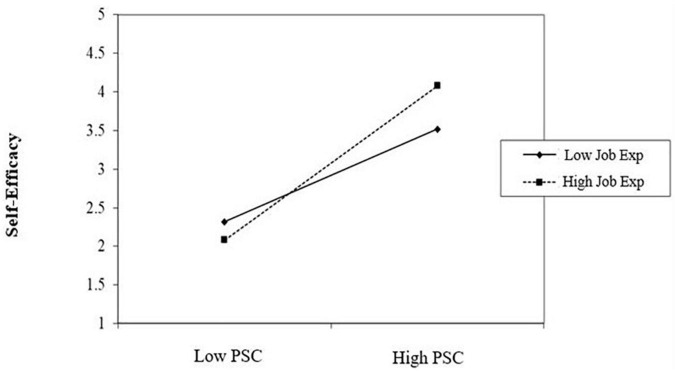
Simple slope analysis for interaction effects of job expectations with psychosocial safety climate scale and self-efficacy. PSC, psychosocial safety climate; Job Exp, job expectations.

The coefficient values in Table 3.1 show that the job-related expectation has a significant moderating effect on the relationship between the psychosocial safety climate scale (PSC-12) and self-efficacy. The overall model is statistically significant, *R* = 0.97, *F* = 2215.49 (3.00, 277.00), ^**^p < 0.01 (Figure 1). The interaction graph is shown in Figure 2. It reveals that job expectations have a significant moderating effect between psychosocial safety climate and self-efficacy. Employees who have better psychosocial safety climates and high job expectations have higher levels of self-efficacy as compared with employees who have poor psychosocial safety climates and low job expectations. These findings accept the Hypothesis (H2).

**TABLE 3.1:** Predicting moderating role of job expectations in psychosocial safety climate scale, and self-efficacy.

	β	SE	*t*	*p*	LLCI	ULCI
Psychosocial safety climate	0.80	0.11	7.03	0.001***	0.58	1.03
Job-related expectations	0.08	0.11	0.67	0.043*	0.15	0.33
Int-1	0.2	0.06	2.33	0.02**	0.02	0.04

Int-1 product term psychosocial safety climate and job expectations. *p < 0.05, **p < 0.01, ***p < 0.001.

Table 3.2 shows differences between employees having low job-related expectations (*n* = 161) and high job-related expectations (*n* = 120) with respect to self-efficacy (which is the comparison of the extreme ends of the interaction graph shown in Figure 2). The results show that significant differences exist in self-efficacy between employees having low job-related expectations and employees having high job-related expectations with *t* (279) = −5.74, ^***^p < 0.05. While employees having high job-related expectations exhibited higher scores on self-efficacy (*M* = 28.20, SD = 5.69) as compared with employees having low job-related expectations (*M* = 24.51, SD = 5.02) during the COVID-19 pandemic. Cohen’s *d* value of the self-efficacy scale was 0.68 (>0.50), which indicated a large effect size.

**TABLE 3.2:** Results of job expectations-wise comparison of general self-efficacy among private-sector employees during the COVID-19 pandemic.

Variables	Low job-related expectations (*n* = 161)		High job-related expectations (*n* = 120)		*t* (279)	*p*	Cohen’s *d*
	***M***	**SD**	***M***	**SD**			
General self-efficacy	24.51	5.02	28.20	5.69	−5.74	0.001***	0.68

***p < 0.001.

### 4.3. Hierarchical regression

**FIGURE 3:**
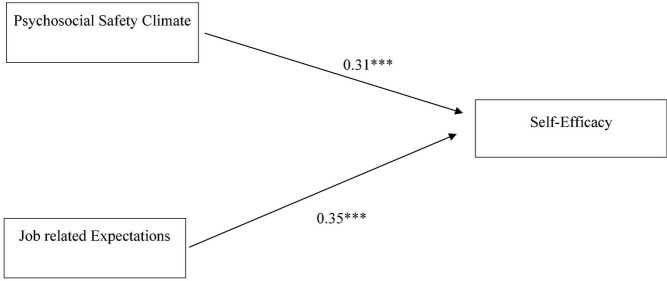
Model showing the direct predictive relationship of psychosocial safety climate and job expectations with self-efficacy. ^***^p < 0.001.

Table 4 shows the results of hierarchical multiple regression. In block 1, demographic characteristics such as age, gender, marital status, and family system were added. The model shows no significance with a *R*^2^-value of 0.02, which implies that 2% variance is explained by the demographics (*F* = 1.05, *p* = 0.39). In block 2, job-related expectations with β = 0.45^***^ show a significant prediction of self-efficacy. The *R*^2^-value of 0.21 revealed that 21% variance is explained by the subscales (*F* = 11.58, ^***^p < 0.001). In block 3, job-related expectations with β = 0.31^***^ and psychosocial safety climate with β = 0.35^**^ revealed significant predictions. The *R*^2^-value of 0.31 shows a 31% variance explained by the subscale compensation and psychosocial safety climate (*F* = 17.14, ^***^p < 0.001). This elicits the direct relationship between IV and the moderator with DC. The predictive model is shown in Figure 3.

**TABLE 4:** Hierarchical multiple regression showing prediction of self-efficacy by demographic variables, psychosocial safety climate, and job-related expectation in private-sector employees during the COVID-19 pandemic.

Variables	Block 1		Block 2		Block 3	
	**β**	**SE**	**β**	**SE**	**β**	**SE**
**Block 1**						
Age	0.05	0.03	0.02	0.03	0.01	0.03
Gender	-0.03	0.69	-0.01	0.62	0.03	0.58
Marital status	0.05	0.72	-0.05	0.67	-0.03	0.62
Family system	0.10	0.72	0.10	0.66	0.08	0.61
Job satisfaction	0.02	0.49	-0.01	0.44	0.01	0.41
**Block 2**						
Job-related expectations			0.45***	0.05	0.31***	0.05
**Block 3**						
Psychosocial safety climate					0.35***	0.04
*R*	0.14		0.45		0.56	
*R* ^2^	0.02		0.21		0.31	
*F*	1.05		11.58***		17.14***	
Δ*R*^2^	0.02		0.18		0.10	

*N* = 281; ***p < 0.001.

### 4.4. Independent sample *t*-test analysis of demographic characteristics

Table 5.1 shows differences between men (*n* = 169) and women (*n* = 112) with respect to variables of the study. The results show significant differences between men and women on psychosocial safety climate scale with *t* (279) = 2.05, *p < 0.05. While women exhibited higher scores on the psychosocial safety climate scale (*M* = 37.83, SD = 8.92) as compared with men (*M* = 35.77, SD = 7.79). Cohen’s *d* value of psychosocial safety climate was 0.24 (<0.50), which indicated a small effect size, partially accepting the hypothesis (H3a).

**TABLE 5.1:** Results of gender-wise comparison of psychosocial safety climate scale, job-related expectations, and general self-efficacy among private-sector employees during the COVID-19 pandemic.

Variables	Women (*n* = 112)		Men (*n* = 169)		*t* (279)	*p*	Cohen’s *d*
	***M***	**SD**	***M***	**SD**			
Psychosocial safety climate	37.83	8.92	35.77	7.79	2.05	0.041*	0.24
Job-related expectations	26.21	7.91	25.13	7.70	1.13	0.257	0.13
General self-efficacy	26.42	5.58	25.86	5.63	0.82	0.410	0.09

*N* = 281; *p < 0.05.

Table 5.2 shows differences between married (*n* = 172) and unmarried (*n* = 109) employees with respect to study variables. The results show a significant difference between married and unmarried on the job-related expectations scale with *t* (279) = −3.70, ^***^p < 0.05. While unmarried exhibited higher scores on the job-related expectation scale (*M* = 27.67, SD = 8.61) as compared with married (*M* = 24.22, SD = 6.91). Cohen’s *d* value of job-related expectations was 0.44 (<0.50), which indicated a small effect size, partially accepting the Hypothesis (H3b).

**TABLE 5.2:** Results of marital status-wise comparison of psychosocial safety climate scale, job-related expectations, and general self-efficacy among private-sector employees during the COVID-19 pandemic.

Variables	Married (*n* = 172)		Unmarried (*n* = 109)		*t* (279)	*p*	Cohen’s *d*
	***M***	**SD**	***M***	**SD**			
Psychosocial safety climate	36.44	8.30	36.83	8.35	−0.38	0.704	0.04
Job-related expectations	24.22	6.91	27.67	8.61	−3.70	0.001***	0.44
General self-efficacy	25.71	5.13	26.67	6.27	−1.40	0.161	0.16

*N* = 281; ***p < 0.001.

Table 5.3 shows the ***t***-test analysis between satisfied (***n*** = 148) and unsatisfied (***n*** = 90) employees with respect to study variables. Results show no difference between satisfied and unsatisfied employees with respect to the study variables, thus rejecting the Hypothesis (H3c).

**TABLE 5.3:** Results of job satisfaction-wise comparison of psychosocial safety climate scale, job-related expectations, and general self-efficacy among private-sector employees during the COVID-19 pandemic.

Variables	Satisfied (*n* = 148)		Unsatisfied (*n* = 90)		*t* (279)	*p*	Cohen’s *d*
	***M***	**SD**	***M***	**SD**			
Psychosocial safety climate	36.48	8.62	36.90	8.56	−0.35	0.720	0.04
Job-related expectations	24.42	7.81	27.26	7.65	−2.74	0.007	0.36
General self-efficacy	25.96	6.00	25.51	5.15	0.59	0.551	0.08

*N* = 281.

## 5. Discussion

The main objective of this article was to find the relationship between psychosocial safety climate, self-efficacy, and job-related expectations. It explored the moderating role of job-related expectations on the relationship between psychosocial safety climate and self-efficacy among employees of private-sector organizations in Pakistan, during the pandemic. It also aimed to explore the differences in married and unmarried employees with respect to the study variables.

Results of statistical analysis show that psychosocial safety climate had a positive significant relationship with job-related expectations and self-efficacy. Job expectations also positively and significantly correlated with self-efficacy. Self-efficacy was also predicted by psychosocial safety climate and job-related expectations. These findings are supported by the available literature. In different organizations, the climate of the organization was found to influence self-efficacy (Tobin et al., 2006; Yi et al., 2008). This is due to the fact that when behavior at the workplace are commended and accepted by others, which is a characteristic of a good organizational climate, they are likely to be repeated, increasing the confidence and in turn, the self-efficacy among the employees, which ultimately benefits the organization (Reaves and Cozzens, 2018; Patras et al., 2020) in the long run as their expectations are also met (Mahal, 2009; Zhang and Liu, 2010; Karantzas et al., 2016).

Moderation analysis showed that job expectations moderate the relationship between psychosocial safety climate and self-efficacy. This is supported by previous research (Jaafari et al., 2012) in which other variables were found to have an impact on the relationship between organizational climate and self-efficacy. Job-related expectations influence the domains or factors associated with organizational climate (Linz and Semykina, 2013; Schiff and Leip, 2018; Sharma and Chully, 2020; Egerová et al., 2021). Once these expectations are met, employees are more likely to have higher levels of self-esteem (Čiarnienė et al., 2010; Zhang et al., 2019). This whole dynamic results in the moderating effect of job-related expectations.

The independent sample *t*-test showed that there are significant gender differences among employees on measure of psychosocial safety climate, with women having higher scores as compared to their male counterparts. These findings are different from the existing literature (Berthelsen et al., 2020; Jane Zadow et al., 2021). This deviation from previous studies can be explained with help of cultural factors. In collectivist cultures like Pakistan, the autonomy and independence of women is constantly undermined by patriarchal values. With increase in awareness and increased participation in non-domestic work, women are paying more attention to the psychosocial climate in an organization for their individual as well as community wellbeing as compared to men who have a sense of innate entitlement.

The independent sample *t*-test showed that there is a significant difference in job-related expectations between married and unmarried employees, with unmarried employees having higher scores on job expectations. No differences were found between the two groups with regard to psychosocial safety climate and general self-efficacy scales (GSE). This is due to the reason that unmarried employees usually have comparatively fewer responsibilities. Their primary focus is their job and they have certain ideals and expectations related to different aspects of the job. They can switch jobs rather easily as they do not have to care about immediate financial aspects and can take risks to meet their job expectations. On the other hand, married employees have other psychosocial needs and responsibilities which they have to attend to. They have to cater to the economic factors as they have dependent members. They look for job security and avoid risk-taking to fulfill their job expectations.

The independent sample *t*-test showed that there are no significant differences between satisfied and unsatisfied employees. This difference is not supported by previous studies (Linz and Semykina, 2013; Huang and Gamble, 2015; Kong et al., 2015; Nie and Sousa-Poza, 2017). This variation can be explained by the change in work dynamics caused by COVID-19 pandemic. The determinants of job satisfaction are different among employees who work physically in office and those who work remotely (Brunelle and Fortin, 2021). The previous research studies are conducted under normal conditions in which employees came to their offices physically, whereas, in the current research, employees were working from home for the first time. As a result, they had different stressors and their individual differences dissipated based on their levels of satisfaction.

### 5.1. Implications

It expands the theoretical framework related to organizational settings during unprecedented situations like pandemics. It helps to understand the organizational strengths and cultural impact on employees during the pandemic. It shares insights to know more about effective paths way to enhance self-efficacy in employees when they face adverse situations that effect their health and general wellbeing.

The current study can be beneficial for Industrial, educational, information technology, and other related organizations in the private sector. Results can be applicable to private-setting employees and organizations across the board, particularly in low- and middle-income countries. The research has implications for improving policies and practices related to organizational climate that ultimately lead to better output and employee satisfaction during unprecedented calamities and stressors. It also highlights the importance of fulfillment of job-related expectations and how they can lead toward satisfaction of employees and ultimately higher participation in organizational tasks and collective achievement of goals, especially in environments where there are looming threats in multiple domains.

## 6. Conclusion

COVID-19 has brought with it elements of shock and economic instability along with other negative repercussions in other areas of life. Organizations and work dynamics were disrupted and altered during the pandemic. The current study explored the relationship between organizational climate, self-efficacy, and job-related expectations along with moderating role of job expectations on the relationship between the other two variables, among employees of private-sector organizations during the COVID-19 pandemic. The findings of the research accepted two of the hypotheses completely, rejected one and accepted the remaining partially eliciting that psychosocial safety climate has a positive significant relationship with job-related expectations and self-efficacy; job expectations also significantly correlated with self-efficacy. Job-related expectations moderated the relationship between organizational climate and self-efficacy. There were significant differences on the study variables based on the demographic characteristics and satisfaction levels of employees. The research highlights the importance of satiation of job-related expectations and cultivation of self-efficacy in employees to increase the economic as well as other outputs of the organizations, particularly during calamities and unprecedented situations. This research provides insight into managing organizational climate and associated factors during stressful situations, health emergencies, and catastrophes.

Economic disparities and psychosocial factors during the pandemic might have affected the results. Only private organizations were selected in the present research and data were not taken equally from different organizations owing to travel restrictions of COVID-19. In the future, the research could be conducted on employees and subjective qualitative aspects of the research area could be tapped. Data can be collected, analyzed, and compared for different age groups, socioeconomic statuses, semi-government organizations, and labor unions.

## Data availability statement

The raw data supporting the conclusions of this article will be made available by the authors, without undue reservation.

## Ethics statement

The studies involving human participants were reviewed and approved by Institutional Review Board, Lahore Garrison University. The patients/participants provided their written informed consent to participate in this study.

## Author contributions

DF: conceptualization, data collection, and initial draft of manuscript. SM: conceptualization, initial draft of manuscript, and finalzing manuscript. FN: statistical analysis, conceptualization, and finalzing manuscript. MS: writing and finalzing manuscript. MN: revising the manuscript critically for intellectual content according to the reviewers’ comments. All authors contributed to the article and approved the submitted version.
